# Rescue blankets as multifunctional rescue equipment in alpine and wilderness emergencies: a commentary

**DOI:** 10.1186/s13049-022-01005-5

**Published:** 2022-03-10

**Authors:** Bernd Wallner, Hannah Salchner, Markus Isser, Thomas Schachner, Franz J. Wiedermann, Wolfgang Lederer

**Affiliations:** 1 Department of Anaesthesiology and General Intensive Care Medicine, Medical University of Innsbruck, Anichstr. 35, 6020 Innsbruck, Austria; 2 Medical Division, Austrian Mountain Rescue Service - Tyrol, Florianistr. 2, 6410 Telfs, Austria; 3 Department of Cardiac Surgery, Medical University of Innsbruck, Anichstr. 35, 6020 Innsbruck, Austria

**Keywords:** Rescue blanket, Hypothermia, Emergency medicine, Wilderness medicine, Bandages, Pelvic binder, Tourniquet

## Abstract

Emergency applications of rescue blankets go far beyond protection from hypothermia. In this review alternative applicabilities of these remarkable multifunctional tools were highlighted. Newly fabricated rescue blankets prove impressive robustness. The high tensile strength along with its low weight enable further applications, e.g. immobilization of injured extremities, splinting, wound dressing, a makeshift chest seal in sucking chest wounds, amongst others. Furthermore, the foil can be used as a vapour barrier, as eye protection and it can even be used to construct a stopgap bivouac sack, as alternative tool for transportation in the remote area and a wind shield or a water reservoir in the wilderness. During search-and-rescue missions the light reflection from the gold surface enhances visibility and increases the chance to be found. Rescue blankets are essential parts of first aid kits and backpacks in alpine and wilderness environment with multifunctional applicabilities. In this commentary to a review we want to evaluate the numerous applicabilities of rescue blankets in the treatment of emergencies by wilderness medicine and pre-hospital EMS.

In this commentary to a structured review on the role of rescue blankets in wilderness medicine and pre-hospital emergency medical services (EMS) the authors want to discuss the numerous applications of this remarkable tool. Originally the aluminum coated polyethylene terephthalate foil was developed by the National Aeronautics and Space Administration’s Marshall Space Flight Center to protect exterior surfaces of spacecraft. In humans the blanket was first used to avoid hypothermia in athletes after marathon competitions [1]. Nowadays, rescue blankets are medical devices category 1 according to directive 93/42/EEC and are essential rescue equipment primarily used for prevention of hypothermia in out-of-hospital emergencies [2, 3].

Freeman et al. reported that blankets or wraps were used by 93% of lowland rescue teams, 85% of lifeguard organisations, 82% of ground ambulances, 71% of air ambulances and 50% of mountain rescue teams [4]. A convincing advantage of rescue blankets is their low-weight and low-bulk properties that take up very little space. Prevention and treatment of hypothermia is achieved by reducing heat loss from convection, conduction, evaporation, and thermal radiation. Zasa et al. observed that different conventional blankets, e.g., space blankets, bubble wrap, blizzard blankets, ambulance blankets and ready heat blankets, significantly reduced heat loss but could not completely compensate for the temperature deficit [5]. Reflective metallic foil blankets had better thermal insulation properties when compared to various blankets during different wind conditions [6]. Beyond that, the physical properties of the aluminum coated polyethylene terephthalate foils indicate an extraordinary potential of alternative applications which make them multifunctional tools. We recently assessed breaking strength and elongation of two common brands of rescue blankets using a tensile strength testing machine [7]. Rescue blankets proved to be strong enough to function as triangular arm-sling and figure-of-eight bandages for immobilization and as alternative tool for transportation in the remote area [7]. Rescue blanket even can be used for circumferential compression as makeshift pelvic binder in pelvic fracture and as tourniquet in bleeding emergencies of the limbs [8]. Whenever rescuers run out of commercial tourniquets rescue blankets may serve as improvised tourniquets for control of severe extremity haemorrhage [9, 10].

Rescue blankets stand out for their protective properties. The silver side of the blanket is highly reflective and flashes in the sun but gold up increases a victim’s visibility in a snow and glacier environment. We assessed transmissivity and reflectivity of electromagnetic radiation from the metallized surface of the blankets using a lens analyzer [11]. Injury to eyes and skin may arise from high-energy rays in the ultraviolet (UV) spectrum and high-energy visible (HEV) light in the violet/blue band during mountain hike. Optometric measurements revealed that rescue blankets can sufficiently block ultraviolet radiation. Thus, rescue blankets can serve as makeshift sunglasses on the glacier [8]. Thermographic imaging by radiometric thermal camera verified that protection from hypothermia to result mostly from the reflection of infrared radiation [12]. Rescue blankets revealed opposed properties. They can facilitate detection of victims in search and rescue missions and they can render detectability impossible. On the one hand, reflection of daylight from the metalized surface enhances visibility. On the other hand, reflection of infrared radiation below the blankets hampers detection search and rescue missions with night vision devices [12].

In the prehospital setting the use of non-occlusive chest seals is essential according to the ERC guidelines [13]. Whenever non-occlusive dressings are not available, makeshift chest seal of polyvinyl chloride packing may be used by first aid providers [13]. In an experimental study we assessed the fitness of a rescue blanket as a provisional seal for penetrating chest wounds in a new ex vivo porcine model [14]. We could show, that the smooth and moist surface of the blanket provides adequate tightness when applied as makeshift chest seal in sucking chest wounds [14]. Even more, the watertight and windproof foil can be used as a rain shield, a wind shield, a stopgap bivouac sack, and a water reservoir in the wilderness [15]. There are more properties of this multifunctional tool worth to be investigated, e.g. electromagnetic properties and characteristics of the surface textures that could determine the role of rescue blankets to protect from aerosols as barrier during cardiopulmonary resuscitation. And the list of further properties to be investigated is not ending.

## Summary

Rescue blankets are essential equipment of emergency kits. Properties and scope of application exceed by far protection from hypothermia and enhanced visibility. In this review we liked to highlight alternative applicabilities of these remarkable multifunctional tools. Newly fabricated rescue blankets prove impressive robustness. We investigated rescue blankets as triangular arm-sling and as figure-of-eight bandages, rescue blankets as makeshift pelvic binder and tourniquet in bleeding emergencies, rescue blankets as makeshift chest seal in sucking chest wounds, rescue blankets as sunglasses on the glacier and as alternative tool for transportation in the remote area.

## Data Availability

The datasets used and analysed during the current study are available from the corresponding author (BW bernd.wallner@i-med.ac.at) on reasonable request.
